# Cardiac Computed Tomography: Application in Valvular Heart Disease

**DOI:** 10.3389/fcvm.2022.849540

**Published:** 2022-03-24

**Authors:** Kush P. Patel, Sebastian Vandermolen, Anna S. Herrey, Emma Cheasty, Leon Menezes, James C. Moon, Francesca Pugliese, Thomas A. Treibel

**Affiliations:** ^1^Barts Heart Centre, St Bartholomew’s Hospital, London, United Kingdom; ^2^Faculty of Population Health Sciences, Institute of Cardiovascular Sciences, University College London, London, United Kingdom; ^3^William Harvey Research Institute, Queen Mary University of London, London, United Kingdom; ^4^Institute of Nuclear Medicine, University College London, London, United Kingdom; ^5^NIHR Biomedical Research Centre, University College London Hospitals NHS Foundation Trust, London, United Kingdom

**Keywords:** valvular heart disease, aortic stenosis, TAVR, TMVR, cardiac computed tomography

## Abstract

The incidence and prevalence of valvular heart disease (VHD) is increasing and has been described as the next cardiac epidemic. Advances in imaging and therapeutics have revolutionized how we assess and treat patients with VHD. Although echocardiography continues to be the first-line imaging modality to assess the severity and the effects of VHD, advances in cardiac computed tomography (CT) now provide novel insights into VHD. Transcatheter valvular interventions rely heavily on CT guidance for procedural planning, predicting and detecting complications, and monitoring prosthesis. This review focuses on the current role and future prospects of CT in the assessment of aortic and mitral valves for transcatheter interventions, prosthetic valve complications such as thrombosis and endocarditis, and assessment of the myocardium.

## Introduction

Interest in valvular heart disease (VHD) has been invigorated with the advancement in new imaging modalities and pathological insights, and most importantly the advent of transcatheter valve interventions. Transcatheter aortic valve replacement (TAVR) has now overtaken surgical aortic valve replacement (SAVR) in volume in Germany (and the United States) (1) and is driving innovation in transcatheter interventions on other valves (2). Cardiac computed tomography (CT) has become an essential tool for the heart valve team to supplement the assessment by echocardiography, and decision making for suitability and mode of intervention. Technical developments in CT technology have made this possible by providing high temporal, spatial and contrast resolution for imaging one of the most challenging imaging targets of the body. The use of CT is now recommended in guidelines for the pre-procedural work-up for TAVR (3) and is an important tool for the diagnosis of valvular thrombosis (4) and infective endocarditis (5). This has led to its widespread use in the assessment and management of patients with VHD providing additional novel insights into remodeling, pathophysiology, and prognosis. This review article explores its current role, limitations and future prospects in the assessment and management of patients with VHD. It does not cover the technical aspects of CT data acquisition and reconstruction, which can be found elsewhere (3, 4, 6).

## Native Aortic Valve Assessment

Aortic stenosis (AS) is the commonest type of VHD in the developed world (7). Treatment using either SAVR or TAVR is considered for severe AS (8). Determining severity is largely done using echocardiography, with aortic valve area (AVA) being the most commonly used marker of severity. It is calculated using the continuity equation with the incorrect assumption of a circular left ventricular outflow tract (LVOT) (9). Although using a CT derived LVOT area for the continuity equation is more accurate than a 2D echo-based LVOT area, this has not translated into better diagnostic performance (correlation with transvalvular gradients) or mortality prediction (10). However, discordant echocardiographic parameters (discordant AVA and gradient) occur in up to a third of patients, making the quantification of AS severity difficult (11, 12). CT has an important role in determining severity among these patients, especially those with paradoxical low-flow, low-gradient AS (8, 13). Calcification is the cornerstone underlying the pathophysiology of AS in most patients. A sequence of pathological changes involving lipid infiltration of the valve, inflammation, fibrosis and mineralization, leads to AS (14). Using a non-contrast CT, calcification is identified as areas of increased radio-opacity. The commonly used Agatston score method defines calcification where the density is greater than 130 Hounsfield units (HU) (15). CT derived aortic valve calcium score (AVCS) demonstrates high inter- and intra-observer reproducibility (16), correlates well with the severity of AS determined by echocardiography (17, 18) and calcium weight on explanted valves (16), thus making it a very useful marker of AS severity. AVCS is also prognostically important (13, 19) and determines progression of AS, with higher AVCS at baseline correlating with faster progression of AS (20). Compared to men, women have less calcification, but more fibrosis for the same severity of stenosis (21), leading to different recommended thresholds for the definition of severe AS; 1,200 Agatston units (AU) in women and ∼2,000AU in men (22). However, these thresholds may not be applicable in patients with bicuspid AS or rheumatic valve disease due to differences in pathophysiological mechanisms (12).

An alternative method utilizes planimetry of the orifice during systole. This anatomical, rather than functional measurement, correlates poorly with other measurements of AVA and with transvalvular gradients (10). Consequently this is seldom used clinically.

Outcomes in patients with moderate AS are known to be poor, especially if systolic function is compromised (23, 24). An ongoing trial is evaluating whether TAVR has a role in such patients (25). CT may play a role in the future for identifying patients for intervention with less than severe valvular disease as calcification has been shown to correlate with the rate of progression and mortality in patients with less than severe AS (26).

## TAVR Planning

The utility of TAVR has seen a dramatic increase over the last decade, with CT being routinely used to facilitate its use, improve efficacy and reduce complications.

### Access Planning

**FIGURE 1:**
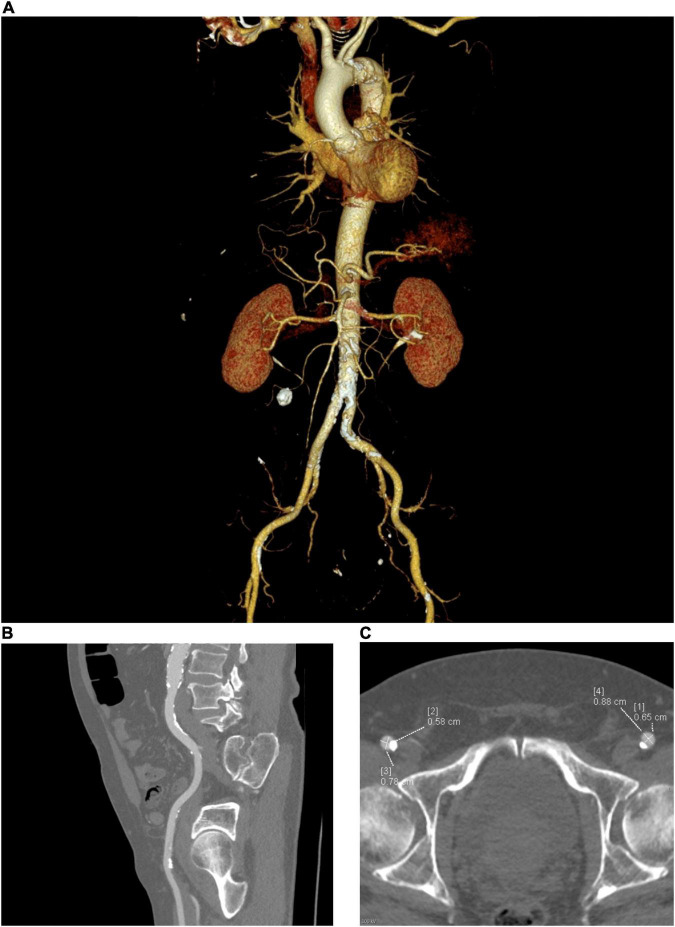
Peripheral access planning for TAVR, requires assessment of the size, tortuosity, calcification (both severity and distribution) and any prosthetic material such as stents or pathologies such as aneurysms. **(A)** Multiplanar reconstruction of the vascular tree, **(B)** Sagittal view, **(C)** Axial view.

Cardiac computed tomography angiography of the aorta and peripheral vasculature provides a quick and complete dataset for TAVR planning (Figure 1). In addition to illustrating the dimensions of the aortic annulus and root, and degree and distribution of aortic valve calcification, CT can demonstrate the degree of iliac vessel wall calcification, tortuosity, ilio-femoral stenosis, presence of aorto-iliac aneurysms, foci of dissection, large penetrating ulcers, and potentially thrombi, as well as previous vascular procedures with grafting/stent implantation—useful considerations for procedural planning (27–29). When a transfemoral access site is unfavorable, CT can provide valuable information regarding alternative sites, such as subclavian, carotid, apical, trans-aortic, and trans-caval (crossing from the inferior vena cava into the abdominal aorta and using a closure device to plug the aortic wall after implantation of the valve) (30). Trans-caval access is increasingly being used and greatly benefits from pre-procedural planning using CT. Using electrocautery, a puncture is made from the inferior vena cava (IVC) to the adjacent descending aorta between the aortic bifurcation and renal arteries. A calcium-free window on the aorta adjacent to the IVC needs to be located using CT and defined by the vertebral level. Additional measurements such as the distance between aorta and IVC, lumen diameters and identification of bail-out access (in case endograft therapy is required) are useful and can be performed using CT (31, 32).

### Implantation Planning

**FIGURE 2:**
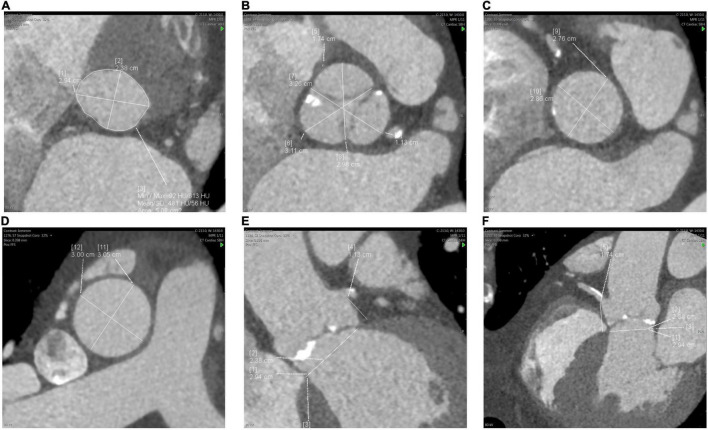
Measurements of the aortic root and ascending aorta. **(A)** aortic valve (AV) annulus, **(B)** sinus of Valsalva, **(C)** sino-tubular junction, **(D)** ascending aorta, **(E)** left coronary ostial height from AV annulus, **(F)** right coronary ostial height.

First, using multiplanar reconstructions (MPR), CT can be used to determine the optimum fluoroscopic projection for valve implantation-orthogonal to the aortic valve (29). This has been shown to reduce additional aortograms, procedural time and contrast use (33). Second, CT provides an accurate guide for sizing an aortic bioprosthesis based on aortic valve (AV) annular dimensions, with a resulting reduction in post-TAVR aortic regurgitation (34, 35). Annulus diameters, area and perimeter are typically used to derive the most appropriate transcatheter valve diameter, applying recommendations provided in manufacturers’ charts. Third, additional measurements are typically taken at levels of the sinus of Valsalva, sino-tubular junction, ascending aorta and the heights of the coronary ostia from the AV annulus- guiding the procedure and enabling the prediction of complications (Figure 2). Low coronary ostial heights and narrow sinuses of Valsalva are associated with a higher risk of coronary obstruction and difficulty in coronary artery engagement for angiography or intervention (36, 37). Correct valve sizing to prevent oversizing is essential to prevent annular rupture, which often results in fatal outcomes (38).

### Predicting Complications

Aortic valve calcification is important to ensure the anchorage of the bioprosthesis and prevent valve migration (aortic root dilatation and the lack of calcification commonly preclude the use of TAVR for aortic regurgitation) (39). However, both an increased burden and bulky eccentric calcification can result in inadequate valve apposition, leading to paravalvular regurgitation, which is poorly tolerated post-TAVR and associated with poor outcomes (40–42).

Conduction abnormalities and permanent pacemaker implantation rates remain high (43). Pacing in TAVR patients is associated with less recovery of left ventricular ejection fraction and a higher rate of heart failure hospitalization (44). Therefore attempts to avoid conduction abnormalities and subsequently pacemaker implantation are important. Device landing zone calcification can predict post-TAVR pacemaker requirement, especially if calcification is located around the LVOT, the basal septum (45, 46) or the mitral annulus (47). Conduction abnormalities can also arise due to a short membranous septum. The bundle of His runs close to the membranous septum and is susceptible to compression by the implanted bioprosthesis. Membranous septal depth is measured from the AV annulus to the start of the muscular interventricular septum. Depths of <7.8 mm are predictive of high degree atrioventricular block (46).

Calcification in the LVOT, especially below the non-coronary cusp, is associated with annular rupture (area under the ROC curve 0.81), a complication that often leads to death (48). Other factors associated with annular rupture include device oversizing and post-dilatation (38).

The height of the coronary ostia from the aortic annulus is an important parameter to measure as short heights can result in coronary obstruction from the newly implanted prosthesis (29). Coronary ostial heights are considered low if <12 mm. The sinuses of Valsalva that house the coronary ostia are also important when considering coronary occlusion. A mean diameter <30 mm is associated with increased risk as the space between the bioprosthetic valve and coronary ostia is reduced (3, 37). However, these cut-offs have low specificity and are not prohibitive for a TAVR. Additionally, CT allows evaluation of the extent and severity of coronary artery disease, which dictates the need for further assessment and management (3, 49).

## Native Mitral Valve Assessment

The mitral valve, annulus and associated apparatus form a complex 3D structure. Although echocardiography remains the primary imaging modality for mitral assessment, CT can highlight valve pathology, provide clues to its etiology and importantly, assist in planning for valve repair/replacement.

### Mitral Regurgitation

Mitral valve prolapse is a common cause of primary MR, and CT can reliably detect this (50). In these cases, two- and three-chamber views can be used to identify leaflet thickening (>5 mm) and a flail leaflet, both seen in the context of mitral prolapse. Using retrospective ECG gating, multiple phases of the cardiac cycle can be reconstructed, facilitating moving cine images, which is important in recognizing prolapse.

In patients with secondary MR, evaluation of the leaflets, ventricle and coronary arteries will enable both the diagnosis and etiology of the MR. In a study of 151 patients with heart failure and functional mitral regurgitation (FMR), CT was able to identify that those with moderate to severe FMR had significantly increased posterior leaflet angles and mitral valve tenting heights at central and postero-medial levels. These were described as the strongest determinants of FMR severity (51). CT can provide accurate left ventricular dimensions enabling an understanding of left ventricular dilatation (52). Other cardiomyopathies can also result in MR. Systolic anterior motion of the mitral valve can lead to MR and has been described with hypertrophic cardiomyopathy and cardiac amyloidosis.

Cardiac computed tomography may also play a role in quantifying MR. When measuring regurgitant volumes in 49 patients with isolated MR, the severity of regurgitation correlated well with echocardiography findings (53). This can be done by calculating total stroke volume of the left and right ventricles (end-diastolic volume minus end-systolic volume), with the regurgitant volume being the difference between the stroke volumes of the left and right ventricle. However, this is rarely used clinically, but could have a role in patients with poor echo windows and if cardiac magnetic resonance imaging is contraindicated.

### Mitral Stenosis

Echocardiography remains the gold standard for the diagnosis and grading the severity of mitral stenosis (MS). However, CT can confirm the presence of related features such as left atrial enlargement, as well as certain appearances, which point to specific causes of mitral stenosis, such as thickening of the mitral valve leaflets with commissural fusion and calcification, commonly seen in rheumatic mitral stenosis (so-called fish mouth appearance) (54).

## Mitral Intervention Planning

Transcatheter mitral valve interventions include technology for both repair and replacement, each requiring different approaches and techniques. An in-depth review of the various technologies available can be found here (55). Once a decision to intervene has been made, CT plays a vital role in procedural planning.

### Annular Dimensions

Sizing the annulus is important for selecting a transcatheter mitral valve prosthesis. The mitral annulus is saddle-shaped. However, for the purposes of certain transcatheter prostheses, a D-shaped annulus can be assumed; the medial and lateral fibrous trigones are connected via a virtual straight line, and the diameter and area then calculated by “tracing” its perimeter border (56). 3D software packages are then able to recreate the annulus, allowing further measurements to be made. Specifically, the landing zone is an important consideration when choosing a transcatheter MV prosthesis; each prosthesis has a different anchoring mechanism and requires certain anatomical characteristics. For this reason, leaflet length, chordal anatomy, the presence of a myocardial shelf and left ventricular cavity dimensions need to be assessed on CT (57).

### Leaflets

The mitral valve has an anterior and a posterior leaflet, both of which have three scallops. These are identifiable via CT, and seen in both reconstructed short-axis and long-axis views. Although echocardiography remains the primary imaging modality to evaluate the mitral leaflets, numerous geometric measurements can also be estimated from CT, including leaflet length, area, tenting height, and coaptation angle. These measurements are important for understanding the mechanism of MR and guiding intervention. Indeed, comparisons of three-dimensional (3D) transesophageal echocardiography and cardiac CT have shown that both imaging modalities provide good detailing of mitral leaflet morphology (58). In addition, calcification and clefts of the leaflets are important to note as these can preclude adequate transcatheter edge to edge repair (TEER) (59).

### Left Ventricular Outflow Tract Assessment

Left ventricular outflow tract (LVOT) obstruction is a known complication of TMVR carrying a significant risk of mortality. A new LVOT (termed the “neo-LVOT”) is formed from the interventricular septum anteriorly and the native anterior mitral valve leaflet. Pre-procedural CT planning simulating a neo-LVOT can help predict the risk of LVOT obstruction (60). Extrapolation from hypertrophic cardiomyopathy studies initially identified a LVOT area of 2 cm^2^ as a safe cut-off for TMVR (61). Further studies specific to TMVR suggested a neo-LVOT < 1.7 cm^2^ at end-systole as high risk (60), but more recent evidence has suggested that even smaller areas are safe (62).

Several factors predict LVOT obstruction; device related, remodeling related and native anatomical factors. Of these, the aorto-mitral angulation is important and can be calculated using CT. Defined as the angle between the mitral annular trajectory and LVOT long axis; the smaller the angle, the lower the risk of LVOT obstruction. Coupled with this, the size of the mitral annulus, annulus-to-interventricular septal distance and LVOT and septal shape should also be taken into consideration (57, 60).

### Mitral Annular Calcification

Mitral annular calcification (MAC) has a reported incidence of between 10 and 42% (63, 64) and in patients with co-existent aortic stenosis is found in 50% of patients (47). MAC can be easily identified and its distribution mapped out using CT. MAC increases the technical complexity of surgical intervention, specifically increasing the risk of AV groove disruption, paravalvular leak and increasing pump and clamp times (65, 66). It also increases the risk for percutaneous intervention; performing TMVR in patients with MAC carries substantially more risk of LVOT obstruction than performing it in valve-in-valve or valve-in-ring (60). MAC can make it challenging to recognize the boundary between annulus and blood pool and therefore accurately measure annular dimensions.

**FIGURE 3:**
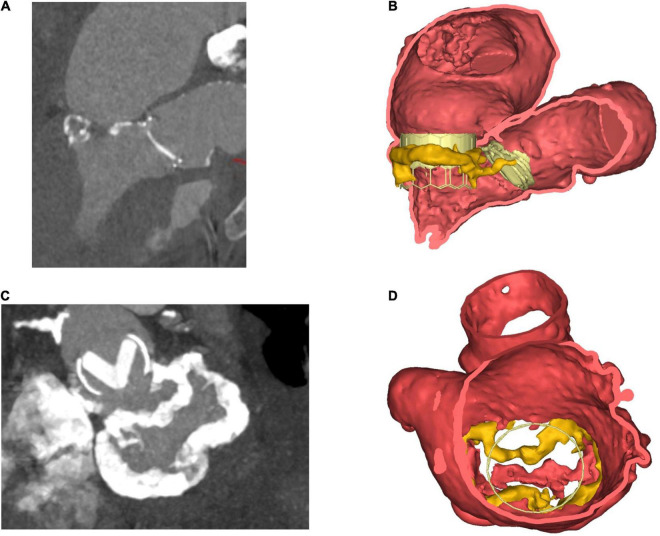
Steps in planning for mitral intervention in a patient with a heavily calcified mitral annulus. **(A)** 2D ECG-gated CT scan. Pre-existing TAVR valve in aortic position, with dense calcification of the mitral annulus. **(B)** Coronal view. 3D volume-rendered image of pre-existing TAVR valve *in situ* in aortic position. Cylindrical valve simulated in mitral position, thereby allowing for anatomical and geometrical calculations to be made prior to implantation. **(C)** 2D CT- En-face view of calcification surrounding mitral annulus. Also visible is the TAVR valve in the aortic position. **(D)** 3D volume-rendered en-face image of mitral annulus down through the left atrium. MAC highlighted in yellow. Panels **(B,D)** created courtesy of post-acquisition processing with Mimics Enlight TMVR planner, Beta version, Materialise NV Inc.

Mitral annular calcification itself can serve as a bed on which the new valve can anchor. In procedures involving the implantation of a TAVR prosthesis in the mitral valve position, non-circumferential or thin MAC can result in poor device sealing (67). MAC can also make it difficult to determine the correct position for valve deployment, with 17% of valve-in-MAC cases requiring a second valve deployed in an early study. The population in this study was at high surgical risk (STS score 14.4 ± 9.5%) and had a 30-day all-cause mortality of 30% (68). In order to plan a TMVR, 3D reconstructions can be created and valve implantation simulated using dedicated off-line software (Figure 3).

### Access Planning

The trans-septal approach is increasingly being used to access the mitral valve. With CT, operators are able to determine the precise anatomy of the left atrium and plan the site of trans-septal puncture in order to minimize the risk of complications, including aortic puncture or myocardial perforation (69). Anomalies within the left atrium that can be seen via CT include aneurysms of the inter-atrial septum, patent foramen ovale and atrial septal defects, all requiring a tailored approach for the trans-septal puncture (70).

With respect to trans-apical approach, access requires an intimate knowledge of the position of the apex and its relation to the chest wall. Valve deployment using this approach requires a perpendicular deployment at the level of the mitral annulus. Once the apex is located, CT can identify the position of the papillary muscles, coronary arteries and chords, so as to plan the approach and prevent complications (61, 71).

As with TAVR planning, CT for TMVR planning can guide vascular access by defining the anatomy of peripheral vessels, including vessel dimension, tortuosity, location and extent of calcification and any prosthetic material such as stents.

### Pre-surgical Planning

Similar to percutaneous mitral valve interventions, minimal-invasive mitral valve surgery (MIMVS) is being performed with increasing frequency. MIMVS commonly refers to procedures involving mini-thoracotomy, port access and robotic-assisted techniques. Certain differences exist between MIMVS and standard open-heart surgery, including access (various locations and lengths of incisions), vision (direct, video-assisted or endoscopic), and cardio-pulmonary bypass strategies (antegrade vs retrograde), as well as between individual centers and operators.

Cardiac computed tomographic angiography and post-processing 3D reconstructions allow assessment for suitability [suitability for retrograde cannulation, presence of a heavily-calcified abdominal aorta, vessel tortuosity and pericardial calcification (72)] and access planning for MIMVS (location of mini-thoracotomy to access the left atrium). CT also allows for evaluation of the aortic dimensions. In MIMVS, one popular technique involves the use of an endo-aortic balloon; its safe use and efficacy dependent on aortic dimensions (73). The CT scan protocol can also include a CT coronary angiogram, which allows for accurate assessment of co-existent coronary disease. This is particularly sensitive in MIMVS patients, many of whom are young with few coronary risk factors and thus low risk profiles for coronary disease (74).

## CT for Guiding Transcatheter Tricuspid Valve Intervention

Cardiac computed tomography also plays a role in the evaluation of tricuspid valve pathology and the planning of related interventions. As with all right-sided lesions, comprehensive assessment via transthoracic echocardiography can be limited by suboptimal cardiac windows, especially when trying to accurately evaluate the three leaflets of the tricuspid valve (anterior, posterior, and septal) and associated structures. Like MR, tricuspid regurgitation (TR) can be primary, but is more often secondary, related to distortion of the right atrial or ventricular anatomy, and consequent annular dilatation.

Cardiac computed tomography facilitates accurate measurement of dimensions for the tricuspid annulus, right ventricular size and distance from the annulus to right ventricular apex, and thus allows deductions to be made as to the likely etiology of regurgitation.

As annular dilatation is often a key pathological process in TR, the majority of devices focus around annuloplasty (including Trialign, Tricinch, and Cardioband), edge-to-edge repair (Triclip and Forma) or the placement of valves in the vena cava to reduce the damage of the tricuspid regurgitant jet on hepatic and renal vasculature (TricValve).

For procedure planning, determining access to the right heart is key. Vascular access can be clearly defined by CT, including vessel dimensions and tortuosity. For the edge-to-edge repair systems, transfemoral venous access is routinely used, whilst for annuloplasty devices a trans-jugular approach is preferred. Accurate assessment of subclavian and axillary veins can also be done, aiding sheath and device delivery (75).

Annuloplasty-based treatments require delineation of landing zones, be that the tricuspid valve annulus, the inferior vena cava or the commissures. The relation of the landing zone with adjacent structures is also important. The right coronary artery runs along the posterior aspect of the tricuspid annulus along the heart’s epicardial surface. Its compression needs to be avoided when securing devices to the tricuspid annulus (76). Calcification along the annulus can also impair percutaneous valve deployment, which can be detected pre-procedurally via CT (77).

## Assessment of Bioprosthetic Valves

### Structural Valve Degeneration

Structural valve degeneration (SVD) is defined as acquired abnormalities affecting the bioprosthetic valve leaflets and/or its supporting structures that eventually results in valve dysfunction (78). One study assessing patients with an assortment of surgical bioprostheses demonstrated at a median follow-up of 10 years, a rate of clinical SVD of 6.6% and subclinical SVD of 30.1% (79). Data on TAVR prostheses have demonstrated a rate of SVD at 1 year of 2.5% (80) and at a median of 5.8 years of 9% with severe SVD affecting <1% (81). CT provides a valuable tool for assessing the structure and mobility of prosthetic valves, but cannot determine transvalvular hemodynamics (78). Therefore the diagnosis and quantification of stenosis or regurgitation is best achieved using echocardiography with CT providing supplementary information.

### Valve Thrombosis

**FIGURE 4:**
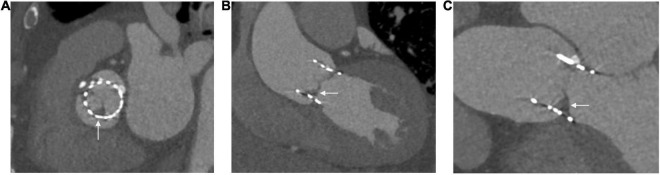
Hypo-attenuated leaflet thickening seen in three views of the same patient **(A)** at the level of the sinus of Valsalva, **(B)** left ventricular outflow tract view, **(C)** three chamber view.

Multi-slice CT angiography has provided important insights into the natural history of prosthetic valves with a particular focus on valve thrombosis. Hypo-attenuated leaflet thickening (HALT) can be found in 10–38% of prosthetic valves (82, 83), with the prevalence possibly higher in TAVR valves (84). Although lacking histological confirmation, this is highly suspected to be thrombus, based on its resolution with anticoagulation (85). HALT usually involves the periphery and bases of a leaflet and extends to a varying degree toward the center of the bioprosthesis (Figure 4) (4). HALT can develop as early as 5 days post-TAVR and has been shown to either progress, stabilize or regress over time (82, 83). Progression of HALT can lead to valve dysfunction described as restricted leaflet motion. This causes an increase in echocardiographically defined transvalvular gradients and eventually leads to symptoms of valve dysfunction (83). CT provides a reliable and potentially more sensitive methodology compared to transthoracic echocardiography for identifying and monitoring HALT (86, 84). It can also help determine management; the composition of acute thrombus has a low attenuation <90 HU, whereas chronic thrombus has values of 90–145 HU. Small acute thrombi are amenable to thrombolysis making this differentiation between types of thrombi important (87). 2D MPR provides an axial cross-sectional assessment to identify leaflet abnormalities. 3D volume rendered CT acquired through multiple phases provides confirmation of the leaflet abnormalities. When reconstructed into a movie (4D virtual reality CT), this can reliably illustrate restricted leaflet motion (4, 88, 89). Prophylaxis and treatment of thromboembolic disease associated with prosthetic valves have important implications for anti-thrombotic therapy, which is discussed elsewhere (90).

Additionally, a common and late complication of prosthetic valves is pannus formation, which commonly coexists with thrombus. Differentiation of the two pathologies is important for management. Pannus has a high attenuation >145 HU and the degree to which it is obstructing the valve orifice can be calculated, making CT a useful modality for its detection (87).

## CT in Infective Endocarditis

**FIGURE 5:**
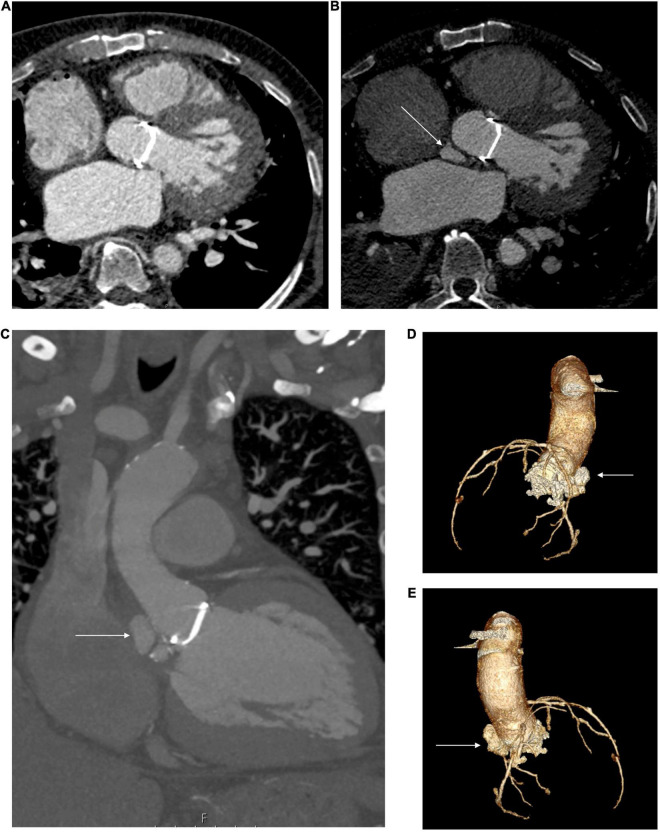
Aortic root abscess seen in a patient with a previous metallic surgical aortic valve implanted in 2010. An axial slice from 2013 without an abscess **(A)**, and a similar slice from 2017 showing a root abscess indicated by a white arrow **(B)**. A coronal view **(C)** and 3D reconstructions **(D,E)** illustrate the abscess indicated by the white arrow.

Although echocardiography is the main imaging modality used to diagnose and monitor infective endocarditis, CT can play a valuable role and its use is advocated by international guidelines (91). CT can provide confirmation of a diagnosis with high accuracy if echocardiography is equivocal. Additionally, CT provides supplementary information such as extra-cardiac foci of infection, abscesses and pseudoaneursyms (5, 92). When combined with positron emission tomography (PET), CT provides diagnostic and prognostic value in prosthetic valve endocarditis for future cardiovascular events (93, 94) (Figure 5).

## Extracellular Volume Quantification (ECV) by CT

**FIGURE 6:**
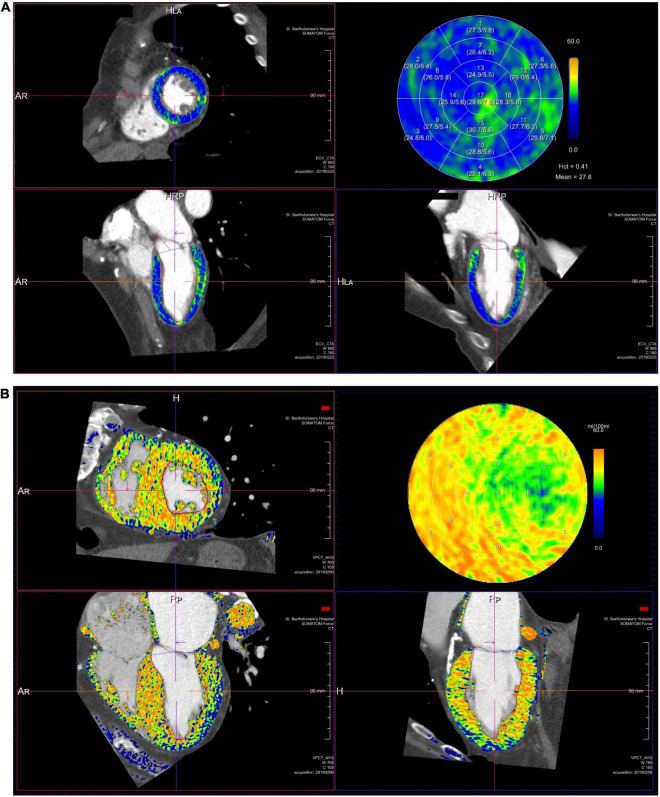
Extracellular volume quantification in two patients: **(A)** severe AS and **(B)** severe AS and cardiac transthyretin amyloidosis. Each panel illustrates a short-axis, 4 and 2 chamber views and a bull’s eye plot. High extracellular volume seen in panel **(B)** is identified by the yellow/orange coloration compared to lower extracellular volume in panel **(A)** identified by the green/blue areas.

VHD causes myocardial remodeling affecting ECV and its composition (95). ECV quantification using cardiac magnetic resonance imaging has been shown to track myocardial fibrosis and provide prognostic value in AS patients (96, 97). Based on similar concepts to cardiac magnetic resonance imaging, ECV can be calculated using CT (98). Certain pathologies such as cardiac amyloidosis result in very high ECV, enabling CT to act as a screening tool (Figure 6) (99).

## Future Applications

Valvular heart disease directly affects the myocardial structure, function, and perfusion. Therefore, assessing these facets guides both, prognosis and management. CT myocardial perfusion imaging (CT MPI) provides prognostic information that can influence management strategies (100). Additionally the quantification of extracellular volume (ECV) has been shown to provide unique insights into diffuse myocardial fibrosis and cardiac amyloid (101). Fusion imaging using a CT overlay on live fluoroscopy imaging may provide a useful tool for transcatheter interventions enabling more complex and safer procedures (102). Photon counting CT potentially heralds a new era in cardiac CT, improving signal to noise ratio, reducing artifacts and radiation. Its integration into clinical use may improve the utility of CT for valvular heart disease (103).

## Conclusion

Cardiac CT has become an irreplaceable adjunct to echocardiography in the clinical assessment of significant aortic stenosis and with the expansion of transcatheter valve intervention, the indications and utility of CT are continually growing. With high spatial resolution, CT allows evaluation of valve anatomy and coronary artery status, aortic pathology and vascular access planning, identification of the risk of likely complications and significant incidental extracardiac findings that influence treatment decisions, prognosis or trigger additional investigations.

## Author Contributions

KP and TT were involved in the genesis and planning of this manuscript. All authors contributed to the literature review, figures, text and editing of the final manuscript.

## Conflict of Interest

The authors declare that the research was conducted in the absence of any commercial or financial relationships that could be construed as a potential conflict of interest.

## Publisher’s Note

All claims expressed in this article are solely those of the authors and do not necessarily represent those of their affiliated organizations, or those of the publisher, the editors and the reviewers. Any product that may be evaluated in this article, or claim that may be made by its manufacturer, is not guaranteed or endorsed by the publisher.

## Abbreviations

AS: aortic stenosis
AVA: aortic valve area
AVCS: aortic valve calcium score
CAD: coronary artery disease
CT: cardiac computed tomography
CTCA: computed tomography coronary angiogram
LVOT: left ventricular outflow tract
MPR: multi-planar reconstruction
MR: mitral regurgitation
SAVR: surgical aortic valve replacement
TAVR: transcatheter aortic valve replacement
TMVP: transcatheter mitral valve prosthesis
TMVR: transcatheter mitral valve replacement
VHD: valvular heart disease.
